# “I value it, but someone should do it”: fitness staff’s perspectives on task-oriented resistance exercise in retirement communities

**DOI:** 10.3389/fpubh.2025.1718945

**Published:** 2026-01-08

**Authors:** Chiung-ju Liu, Lauren Mansuy, Maren Jorgensen, Nichole Stetten

**Affiliations:** 1 Department of Occupational Therapy, University of Florida, Gainesville, FL, United States; 2 Washington University and Barnes-Jewish Orthopedic Center, Chesterfield, MO, United States; 3 Survey Research Center, School of Public Health, Brown University, Providence, RI, United States

**Keywords:** community-based intervention, functional exercise, implementation barriers, late-life disability, progressive resistance exercise, retirement communities

## Abstract

**Background:**

Many older adults relocate to retirement communities to age in place, particularly when facing health declines or disability. Task-oriented resistance exercise, which integrates functional daily activities into exercise, has shown benefits in reducing late-life disability. However, little is known about how such programs can be embedded within the existing fitness infrastructure of retirement communities to support residents who are at risk of disability. This study explored fitness staff’s perspectives on the compatibility and complexity of implementing a task-oriented exercise program in their communities.

**Methods:**

A qualitative descriptive design was applied. Fourteen fitness staff members from 14 various retirement communities across Florida participated in individual semi-structured interviews. Prior to the interviews, participants were provided with a primer on the task-oriented resistance exercise program, as none were previously familiar with it. Interview data were analyzed using inductive content analysis.

**Results:**

Four themes emerged: (1) *whether to screen or not*, (2) *small versus large group formats*, (3) *should work but cannot work*, and (4) *I value it, but someone should do it*. While staff expressed positive impressions of the program and its potential to benefit at-risk residents, these themes reflect instructors’ ambivalence regarding program procedures (e.g., conducting screening), structural considerations (e.g., class size and exercise intensity), scope of practice (e.g., delivering exercise in residents’ homes), and resource constraints (e.g., limited fitness personnel).

**Discussion:**

Fitness staff shared a positive impression of the program and recognized its potential to support at-risk residents. Nonetheless, they also identified several potential implementation challenges, including competing job responsibilities, limited staffing, and the lack of collaboration with healthcare professionals. These findings underscore the need for targeted strategies to address these challenges.

## 1 Introduction

A retirement community is an aggregation of age-restricted housing units within demarcated geographic boundaries purposefully developed for older adults (1). These communities often provide some level of supportive services or leisure amenities. Moving to a retirement community has become a common choice for older adults to age in place. Experiencing failing health, feeling socially isolated, undergoing functional decline, and fearing becoming a burden to family members are major factors affecting the decision to relocate (2, 3). Moreover, amenities in these communities, such as gyms and fitness programs, are an attraction for older adults who want to live an active lifestyle (3, 4).

Although residents in retirement communities vary in health and functional status, recent studies suggest that some may be at risk of disability or long-term care placement. For example, age-related low muscle mass or sarcopenia is a key indicator of frailty and care dependency (5). The prevalence of low muscle mass among residents of the retirement community is higher compared to the nationally representative sample in the United States (6). The prevalence of residents with a frail or prefrail status is 58% in men and 74% in women (7). Compared to non-residents, residents in these communities have more limitations in performing activities of daily living (8). Identifying residents at risk of physical and functional decline and providing timely preventative intervention programs may be beneficial to delay these residents from moving to the next level of living arrangements that offer more costly care services, such as assisted living or long-term care facilities.

The fitness infrastructure in retirement communities increases residents’ access to exercise. The primary responsibility of fitness staff in these communities is to promote healthy aging through physical activity, which can be considered a form of primary prevention in public health (9). The World Health Organization 2020 guidelines on physical activity and sedentary behavior recommend that adults 65 and older should engage in varied multicomponent physical activity at moderate or higher intensity on three or more days a week: 150–300 min of moderate-intensity or 75–150 min of vigorous-intensity (10). However, following these exercise guidelines for residents of the retirement community who already experience a frailty-related functional decline is a challenge.

An intervention program with dual purposes of improving physical capacity and functional ability could be offered to support at-risk residents’ desire to age in place. Fitness staff in these retirement communities could be great candidates to implement the program. Such a program will require the staff to identify residents who are at risk and then offer the program to the selected residents. Harnessing the fitness infrastructure in retirement communities to detect and intervene among residents at risk of late-life disability is considered a form of secondary prevention in public health (9). Note that programs designed as secondary prevention are different from those designed as primary prevention, which usually target individuals with a higher risk.

Prior research has investigated the perspectives of fitness staff who implemented a community-based exercise program targeting adults with balance and mobility limitations in recreation centers (11, 12). The program incorporated a healthcare-recreation partnership (13). Healthcare professionals referred adults with balance and mobility limitations to receive the exercise program from licensed fitness staff who had completed program-specific training. The partnership helps maintain the program’s fidelity and focus on exercisers’ safety. However, such a partnership is not without challenges. Challenges of implementing the program identified by the fitness staff include difficulty coordinating fitness personnel to deliver the program, high cost or inconvenience for exercisers to travel to the recreation centers, and high cost of running the program with a ratio of four exercisers to one staff member. Implementing secondary prevention fitness programs in retirement communities may face similar challenges, worth exploring further.

The major purpose of this study was to understand retirement community fitness staff’s views on a task-oriented resistance exercise program initially developed to improve the performance of activities of daily living in prefrail, community-dwelling older adults. Prior feasibility and field testing results indicate that the program is safe and effective (14, 15). The program has been translated and implemented by two fitness instructors in one retirement community in the State of Indiana in the United States (16). Muscle strength, physical functioning, and performance and satisfaction in activities of daily living were improved in residents who participated in the fitness-staff-led program. Further understanding the perceptions of the feasibility of implementing such a program among fitness instructors who are unaware of the program is informative for scaling up the program and extending the fitness infrastructure in retirement communities to reduce late-life disability. The purpose of this study was to explore the perspectives of retirement community fitness staff on the compatibility and complexity of the task-oriented exercise program after they were newly introduced to it.

## 2 Materials and methods

### 2.1 Study design

A qualitative description research design was applied. Qualitative description is a naturalistic inquiry method using low inference interpretation to understand a phenomenon, a process, or the perspectives of the people involved (17, 18). The approach differs from a traditional lens approach for theory development, i.e., grounded theory. Following qualitative description methods, purposeful and/or convenience sampling was used to recruit participants (17, 18). The Institutional Review Board at the University of Florida approved the study. Informed consent was obtained using the university’s REDCap eConsent system.

### 2.2 Participants

Participants were recruited through the purposive and convenience sampling methods. Internet searches were conducted to identify senior living communities or retirement communities in major cities across Florida. The fitness staff of these communities were then contacted by phone or email to determine their interest and eligibility between September 2020 and May 2021. The research team made up to five contact attempts with each community’s fitness staff before moving on. Fitness staff were eligible to participate in the interview study if his or her job responsibilities involved delivering exercise programs to residents, and if he or she had been employed by the community for 6 months or longer. Fitness staff were excluded if they only delivered specialized exercise programs, such as yoga or tai chi, on an hourly part-time basis.

### 2.3 Procedure

None of the participants had prior knowledge of the task-oriented resistance exercise program. To familiarize them with the program, a primer outlining its general purpose and structure was emailed to each participant after the informed consent process. Participants were instructed to review the primer before their scheduled interview, usually held within 1 week. During the individual interviews, which were conducted via a secure video conferencing platform, participants were asked to respond as if they were considering adopting the program in their own retirement communities.

The primer described the target population as residents at risk of losing independence at home. The program includes resistance band exercises conducted in small groups (up to six residents) in the community gym, and one-on-one activities of daily living exercises conducted in residents’ homes. The program is delivered three times per week over a 10-week period, comprising 24 group sessions and six individual sessions. Each session lasts approximately 50 to 60 min. Additional details about exercise movements and intensity were provided during the interview.

### 2.4 Semi-structured interviews

Interview questions were developed to understand the program’s feasibility, the fitness staff’s work experience and job responsibilities at the current retirement living community, and the gym amenities and fitness programs. Interview questions on feasibility were guided by the Consolidated Framework for Implementation Research (CFIR) (19). Specifically, the feasibility was based on the compatibility (under the CFIR domain of intervention characteristics) and complexity (under the CFIR domain of inner setting) of the program, including: (1) the fitness staff’s acceptance of conducting screenings to select eligible residents for this program, (2) the feasibility of the program duration, frequency, and exercise intensity for the fitness staff and residents, (3) fitness staff’ perceptions of executing the program, and (4) fitness staff’s perceptions of delivering the activities of daily living exercise one-on-one in the resident’s home. Interview guides are available in Appendix 1. The interview duration ranged from 30 to 60 min. Each participant received a $20 gift card as compensation for their time participating in the interview.

### 2.5 Data management and analysis

The audio recording from each participant’s interview was transcribed verbatim and reviewed for accuracy. From the transcripts, textual data related to fitness staff’s work information, the community fitness infrastructure and environment, and residents’ engagement were extracted and summarized. Textual data related to the feasibility of implementing the task-oriented resistance exercise program underwent inductive content analysis (18, 20) using the NVivo software (QSR International Pty Ltd., 2020). In inductive content analysis, the analysis process is driven by the data itself to allow an exploratory approach to understanding the community fitness staff’s perceptions of the feasibility of the task-oriented resistance exercise program. Data saturation was considered when no new information emerged from the participants during data collection (17).

Interviews were analyzed using an iterative coding process between two to three researchers (20–22). Transcripts were first read several times to achieve immersion and to develop a deep understanding. Text data was then read line by line to capture initial thoughts, ideas, and patterns. The first round of the coding process began with identifying and labeling phrases and sentences in the raw data. Each transcript was first coded independently by each researcher. Researchers met regularly to discuss any divergent perspectives during the coding process. Since the researchers’ main interest was to understand the fitness staff’s perceptions of the feasibility of implementing the program, themes that surfaced in the first round of coding were expected to align closely with the questions.

During the second round of coding, the authors examined the coded data more closely and developed themes and subthemes based on participants’ perceptions of program compatibility and complexity (19). Program compatibility can be determined from both conceptual and pragmatic perspectives. The conceptual perspective explored the alignment between the perceived values of the program outcomes and the fitness staff’s own norms, values, and perceived risks and needs. On the other hand, the pragmatic perspective considered the fit between the program requirements (e.g., conducting screening and visiting residents’ homes) and the staff’s workflow, as well as the community fitness structure. Furthermore, program complexity refers to the level of difficulty perceived by the staff when considering the potential of implementing the program in their communities, which is related to the actions and resources required and the types of people targeted by the program (i.e., fitness staff and at-risk residents). Researchers used the constant comparison method to compare themes and subthemes until full consensus was reached (23). The authors continually discussed and compared themes and subthemes to ensure the representation and interpretation of these themes.

### 2.6 Trustworthiness

The rigor of the qualitative analysis was enhanced through repeated coding cycles, constant comparison of the data with the emerging and finalized categories, the use of reflexivity to minimize bias, and peer debriefing with a more experienced qualitative researcher. Additionally, investigator triangulation or peer reviews were used to confirm findings and increase the trustworthiness of results (24, 25).

### 2.7 Positionality statement from the lead author

As a gerontologist focused on late-life exercise and disability prevention, I bring both academic and practical experience to this study. My commitment to supporting age in place through community-based programs informs my perspective. Acknowledging that my role in developing the exercise program may influence the research process, I remain reflective and transparent throughout data collection and analysis to ensure rigor and integrity.

## 3 Results

A total of 110 retirement living communities in the state of Florida were contacted. Of these, 38 did not respond, 33 reported having no fitness staff, and 23 were not interested. Sixteen fitness staff members provided informed consent, but two did not complete the interview. As a result, data from 14 participants were included in the analysis. These participants worked in 14 different individual communities. Table 1 shows the demographic information and work experience of these participants.

**Table 1:** Participant demographics and work experience.

Number	Gender	Age range	Race/Ethnicity	Job title	Length of fitness experience	Length of employment	Employment status
1	Female	50s	White	Fitness/wellness director	30 years	14 years	Full-time
2	Female	60s	White	Fitness/wellness director	40 years	5 years	Full-time
3	Female	50s	White	Fitness/wellness coordinator	17 years	11 months	Full-time
4	Female	40s	White	Fitness/wellness director	17 years	10 years	Full-time
5	Female	50s	White	Fitness instructor	10 years	1.5 years	Part-time
6	Female	30s	White	Fitness/wellness director	17 years	2 years	Full-time
7	Female	60s	Asian	Fitness instructor	35 years	3 years	Full-time
8	Male	20s	White	Fitness/wellness director	5 years	11 months	Full-time
9	Female	50s	White	Fitness/wellness coordinator	Not reported	10 months	Part-time
10	Female	40s	White	Fitness/wellness director	Not reported	6 months	Full-time
11	Male	50s	White	Fitness/wellness director	Not reported	20 years	Full-time
12	Female	20s	Hispanic	Fitness supervisor	3 years	10 months	Part-time
13	Female	50s	American Indian & White	Fitness/wellness coordinator	30 years	9 months	Full-time
14	Female	20s	Hispanic	Wellness associate	Less than 5 years	11 months	Full-time

### 3.1 Summary of the community gym amenities and fitness programs

All 14 communities had at least one fitness gym. Nine provided fitness supervision for their residents within the gym. Six communities utilized fitness measures to track the residents’ baseline fitness levels and improvement, whereas the rest did not have measures in place. Eight communities offered fitness classes ranging from low to high intensity, while the other six offered classes ranging from low to moderate intensity. The length of the fitness classes was between 30 to 45 min. While these communities offered various types of classes, popular classes mentioned by the participants were aqua fitness, Tai Chi, yoga, dance, and muscle strengthening exercise. The average class size was between five and 15 residents in seven communities and between 16 and 30 in three communities. Both ranges applied to the other four communities. In 10 communities, fitness classes operated on a first-come, first-served basis. In two communities, advance sign-up was required, while the remaining two communities implemented a combination of both approaches. Eight communities offered individualized one-on-one fitness programs to their residents.

### 3.2 Summary of residents’ engagement in fitness activities

Participants were asked to estimate the percentage of residents who engaged in their fitness classes or utilized the community gym. Five participants estimated about 5 to 15%, two estimated 20 to 30%, and four estimated 40% or above. Participants also shared strategies to encourage residents’ participation in fitness programs. These strategies included promoting exercise and fitness opportunities in the community, providing a variety of fitness activities, educating residents about the benefits of exercise, and increasing social contact with residents. Ten participants emphasized the importance of motivating and encouraging their residents. They tried to build relationships with their residents through simple greetings and attending other community events.

### 3.3 Feasibility of implementing the task-oriented resistance exercise program

Four themes emerged: (1) whether to screen or not, (2) small versus large group formats, (3) should work but can not work, and (4) I value it, but someone should do it. These themes reflect participants’ ambivalence regarding the program procedures (e.g., program screening and home visit procedures), structures (e.g., class size, exercise types), scope of practice (e.g., delivering exercise in residents’ homes), and resource needs.

#### 3.3.1 Theme 1: whether to screen or not

The theme of “whether to screen or not” describes participants’ opposing views on screening residents for the task-oriented resistance exercise program, which targets those with muscle weakness and difficulty in daily activities. While all acknowledged that some residents met these criteria, opinions varied on the value and consequences of screening.

Some recognized the benefits of screening. Screening could be a good mechanism to match residents with the right program.

*I would think that, that (screening) might be good, because then you can, you can place them more appropriately into the level of intensity for that class. (Participant #11)*.

Being clear about the screening purpose increases residents’ acceptability, and matching residents to the needed program also takes residents’ safety into consideration.

*I mean, I think it's all about how you approach the subject with them. For us, we always start with explaining the importance of the assessment, or the screening, or whatever you're using to kind of rule people out or determine if they're a candidate. So, I always stick to, what is the main reason why we're doing this, okay, for your safety. (Participant #8).*

However, some participants did not see the value of screening and were concerned about excluding residents who were too fit in the independent living units, and feared it could discourage gym participation. They considered the program better for residents in the assisted living units.

*The point is, I guess, you know, if you're going to have a fitness program, you should welcome anyone in for whatever reason… Eventually, what would happen if they, if they're, let's say, they're more able than the level of the class (program), they'll phase out, they'll just won't go (to the gym) anymore, you know (Participant #2).*

*I would think that would be our assisted living mainly. Especially when you're talking about activities of daily living. That's why they moved to assisted living, is because they need help with that (activities) (Participant #3).*

Participants expressed hesitations in rejecting residents from joining the program if they turn out to be ineligible. They suggested communicating the screening results in a positive way to encourage residents to stay active after being screened out of the program, especially immediately after encouraging them to complete the screening. If residents exceed the program requirements, encourage them to stay active by joining other programs. If residents are below the program requirements, encourage them to contact the health care therapy services offered onsite.

*Instead of just saying, 'oh, you're not a good fit for that.' Because then that's like a big bummer and a let-down, especially if they've just gone through a whole screening… I would have something positive and say, 'hey, I think you might be a really good fit for such and such.' (Participant #4).*

*If I were to tell someone they're not eligible for the program, I would probably put a positive spin on it…I would offer them something else. They're excluded from this (program) but they can have that (other options) (Participant #9).*

*If they're too well fit, just say 'keep doing what you're doing, you're doing great.' If they're under fit, maybe, reach out to the therapy team, and let them (residents) know items they can work on. I've been taught in the years of doing this— you have to always have options. (Participant #10).*

#### 3.3.2 Theme 2: small versus large group formats

The theme of “small versus large group formats” represents participants’ mixed views on the small group or one-on-one delivery format in the task-oriented resistance exercise program. The program includes group resistance exercises (three to six residents per group) in the gym, which was praised for enabling personalized feedback, better supervision, and fostering social connection.

*That's definitely going to help with providing more feedback to them biomechanically. If you're working with only four or six people, you have the opportunity to give some very specific direction to each one of those people (Participant #1).*

*So that's something we see with our group…, which is about the same number, about five or six. And a lot of times they start to have kind of friendships or make friendships and kind of help push each other and socialize a little bit more. And that's what makes it a little bit easier to do group training or show up for sessions like that, where you have somebody else helping to keep you accountable, so, outside of the instructor (Participant #8).*

The task-oriented resistance exercise program also includes activities of daily living exercise, delivered one-on-one at the resident’s home. The format allows the instructor to individualize activity exercises, helping residents link the benefits of resistance exercise with activities essential for their independence at home. The format would also be convenient for at-risk residents who have difficulty leaving the home.

*Well, I think the benefits are, you're going to be providing the resident an opportunity to see that change in real, measurable terms, right? Because if they start out with struggling with some of those ADL (activities of daily living), and by the end of the program, they feel much more confident and competent, that's huge (Participant #1).*

*I think the more sedentary or the more pain that a client(resident) has, or if they start to have kind of a, if they can't walk well and they have to use a walker or a cane, they start to almost seclude themselves more and more because they get frustrated by having to use it (walker or cane). And they know they're starting to slow down and they just can't go as far… And so, having somebody go into the home and assist with that or show them is going to make their compliance that much higher (Participant #8).*

*When you're dealing with that, more frail, older population, where just getting dressed and getting out of the house and going is a pretty major commitment, you know, for them to do that. Somebody coming to them would be very well received (Participant #2).*

However, participants also highlighted the logistical and financial challenges of maintaining small groups and one-on-one sessions. More attendees per class would be the best use of the fitness staff’s time.

*Because it's only my assistant and I teaching, we either have to be in the fitness center monitoring the residents. Or if we're teaching, our time is very precious, so we want them, the more residents in each class (Participant #3).*

*I just like to have as many people in my classes I possibly can, because then it lets me know that there's more people that are benefiting (Participant #13).*

The limited staffing also challenges the feasibility.

*Yeah, I just, I don't think it's something that just activities, like the activity department could get done…It's just too small. It's too underfunded. It's stretched too thin… (Participant #6).*

*Um, no, I don't think that's feasible… I do need eyes on the gym, and that's my responsibilities. I can't afford six hours out of my day for that (one-on-one activities of daily living exercise) (Participant #14).*

#### 3.3.3 Theme 3: should work but can not work

The theme of “should work but can not work” reflects participants’ conflicting views on the exercise duration, intensity, and frequency. Participants acknowledged that the program’s structure—three one-hour sessions per week over 10 weeks at moderate intensity—was appropriate for achieving meaningful functional and fitness outcomes. They emphasized that sustained engagement is necessary to see measurable improvements.

*You have to have a realistic time frame if you want to see some real, measurable gains, right? In strength and functional ability, you have to, you have to make an investment of at least that amount of time (Participant # 1).*

*Three times a week for an hour? Yeah. I think they might feel, maybe, overwhelmed at first…I think the amount of time would be a good amount of time to accomplish what it is you're trying to accomplish (Participant #5).*

On the contrary, participants were concerned that residents who meet the program eligibility criteria may not be able to follow the exercise regimen. They questioned whether frailer individuals could tolerate an hour-long session or maintain consistent attendance due to health or personal barriers.

*A lot of residents have a hard time committing, um, to come in and, also, I feel like the hour, hour-long class is just a little too long, I feel like their attention span, and you know, their physical span will be like 30 minutes max. So, I feel like an hour would just be too long (Participant #14).*

*Specific appointments that they might have other than being at your exercise class were a doctor's appointment…sickness. You know, if they get sick, they don't feel good that day. Medication problems, families in town. In other words, families are going to trump your workout (Participant #11).*

#### 3.3.4 Theme 4: I value it, but someone should do it

Theme 4 “I value it, but someone should do it” reflects the staff’s hesitation when considering delivering the one-on-one activities of daily living exercise at the resident’s home, despite recognizing the purpose of the exercise. The purpose of the activities of daily living exercise is to improve residents’ functional performance, which aligns with their job role as fitness staff.

*I think making those tasks easier for them to perform as time goes on. And then for the instructor just being able to see the improvements is always very fulfilling to actually see that you're making a difference in someone's life (Participant #5).*

*They (residents) can see the use of it (exercise) in the home. Right, so I get to see how they, they are exercising in the home… how they can use their, you know, what they have around them to functionally work better (Participant #9).*

*'That would be really beneficial to them (residents), because some of them actually have to hire somebody to clean their homes or to do their laundry, because it is difficult for them, or it is hard for them (Participant #7)’.*

*Well, I think the benefits are, you're going to be providing the resident an opportunity to see that change in real measurable terms, right? Because if they start out with struggling with some of those ADL (activities of daily living), and by the end of the program, they feel much more confident and competent, that's huge (Participant #1).*

*A lot of them (residents) move in because they struggle with their ADLs (activities of daily living). So, working on ADLs would lessen their, their need of help, would give them a sense of freedom (Participant #10).*

Despite this shared appreciation, many participants were hesitant to take on the responsibility of delivering these exercises themselves. Some noted that many residents outsource household tasks and may not see the relevance.

*Um, just because most of the residents don't do their chores. They hire people to do so. And there just seems to be a negative connotation with like doing chores (Participant #12).*

Some participants preferred helping residents connect exercise movements with daily activities in gym-based exercise rather than delivering activities of daily living exercise at the resident’s home.

*That's not really my forte… If we have dumbbells or the resistance bands and we're doing like an overhead press, I say, 'okay, it's like you're putting that box on top of the refrigerator, you got to reach up push' you know… So, I do use activities of daily living in my vocabulary when I'm having them perform an exercise (Participant #3).*

Logistical and structural barriers also emerged. Participants noted that their roles are centered in the gym and focused on encouraging residents to engage in gym-based exercise.

*We tend to make it a habit not to go to their apartments and do one-on-one…Because the whole idea is to get them out of their apartment… (Participant #13).*

Additionally, there may be a need for a change of job description or hiring additional personnel in order to deliver home-based exercise.

*I would have to make some structural changes in their job descriptions because both my full-time personal trainers also have supervisory responsibilities in the fitness center, so they really can't leave to go into residents' homes. So, we would have to hire other personal trainers that would take that on (Participant #1).*

Finally, concerns were raised about safety and the scope of practice, especially when working alone with frail residents.

*They're (staff) not a medical person… a nurse, a home nursing care person or… And now they're in a situation with somebody pretty frail, and they're in their home, and that they (staff) don't have any backup or support in that environment should anything unusual occur. So, I think that would be a little bit of a fear on the part of the instructional staff, saying 'I don't feel, you know, I feel a little bit uneasy with this, this person (resident). Because maybe they (residents) have some concerns or issues or, you know, perhaps they don't breathe well or you know any of those things could be a concern. So, that sort of just me and you alone in this space, you know, 'do I feel like I'm qualified?’ (Participant #2).*

*That's something that our occupational therapist would do…Um, so I would be very cautious about having somebody who is not an OT (occupational therapist) doing those types of things (Participant #4).*

## 4 Discussion

The feasibility of the potential to implement the task-oriented resistance exercise program in retirement communities was explored from the perspectives of compatibility and complexity. The program aligns with the fitness staff’s values of promoting physical exercise to support residents’ independence. Staff acknowledged the importance of screenings to identify at-risk residents, the need for resource-intensive instruction, and the inclusion of daily living activities as exercise. However, the program’s practicality is challenged by limited fitness personnel, their defined scope of work, and their comfort level when working alone with at-risk older adults. This tension between the ideal and the practical is reflected in the four identified themes.

Some fitness staff support the idea of screening residents to identify those at risk of developing disabilities. However, resistance to implementing this screening process also exists. Excluding residents from a program conflicts with their primary responsibilities of promoting physical activity within the community. Their commitment to encouraging physical exercise aligns closely with the principles of primary prevention in public health (9). Redirecting overqualified residents to alternative programs may help fulfill their job responsibilities if a screening procedure is implemented.

Resistance exercise is widely recommended to increase muscle strength, reduce frailty, and improve physical functioning in older adults (26). However, fewer than 25% of older adults meet the recommended exercise guidelines of engaging in resistance exercise twice a week (27). Social support and encouragement from peers and gym staff are key motivators for older adults to participate in resistance exercise (28). Moreover, social cohesion plays a crucial role in increasing older adults’ participation and adherence to exercise classes (29). Fitness staff in this study identified similar motivators when they commented on the small-group resistance exercise. The small group format is perceived to foster social interactions among residents and allows for better supervision.

Most fitness staff expressed concerns about the resource-intensive instruction format. A key aspect of their role is welcoming residents to group fitness classes in the community gym, where greater participation allows them to maximize their time and effort. However, delivering the small-group resistance exercise and one-on-one activities of daily living exercise would stretch current fitness personnel. Similar concerns about personal constraints were noted in a community-healthcare partnership program that implemented a small-group exercise program for adults with balance and mobility limitations (13). Providing adequate support to fitness personnel would be critical for successfully implementing the task-oriented resistance exercise program in retirement communities.

Additionally, fitness instructors’ job routines typically do not include providing individual exercise in residents’ homes. Some participants in the current study felt unprepared to manage residents’ health needs if they were working alone with at-risk residents. Allied health professionals, such as occupational therapy practitioners, may be better suited to deliver the one-on-one activities of daily living exercise. Similar concerns have been documented in the literature regarding instructing exercise for individuals with chronic conditions or disabilities. In a prior study, fitness instructors reported a lack of knowledge and training in supporting people with chronic conditions to exercise (30). They were uncertain about the proper exercise intensity for these individuals and might seek advice from experienced peers. Another study reported a similar gap in knowledge and training for fitness professionals working with people with disability (31). The study recommended collaboration with allied health professionals to “upskill” fitness professionals. However, the primary concern expressed by the fitness staff instructors in the current study is more about being alone at the residents’ homes rather than instructing the residents in activities of daily living exercise. Establishing emergency response procedures may instill confidence and preparedness to deliver the program.

Illness or poor health is a well-known barrier to exercise for older adults (32). While participants acknowledged that the intensity, frequency, and duration of exercise are appropriate to achieve the desired outcomes, they noted that residents’ attendance is likely to be affected by doctors’ appointments or health issues. Additionally, some staff instructors expressed concern that at-risk residents might struggle to tolerate sessions lasting longer than 30 min. This concern could be alleviated by sharing prior feasibility studies showing that the program’s dosing is well-tolerated by pre-frail older adults (14–16). At-risk residents may also become more committed to the program after a screening process and being informed of the program schedule in advance. Furthermore, the program’s design includes a gradual progression, with the first week focused on helping residents become familiar with the exercises before progressively increasing the intensity to a moderate level.

Being able to take care of themselves and do things they like without relying on others is central to older adults’ perceptions of successful aging (33). Fitness staff perceived that incorporating activities of daily living into exercise would help residents recognize improvement in their independence. They expected that residents would notice these improvements as they progress with the program. However, some staff noted that residents might not find the idea of turning daily tasks into exercise appealing. Older adults may prioritize leisure activities over daily living tasks (34). Additionally, supportive services or amenities, such as housekeeping and dining, may be why older adults choose to relocate to a retirement community to enjoy a more carefree lifestyle. Consequently, the task-oriented resistance exercise program may not resonate as strongly with these residents, whose primary interest is in leisure activities.

Some fitness staff preferred not to deliver this part of the program. They considered that verbal instructions could help residents link gym exercise movements with daily tasks. A prior study has shown that such instructional education improves older adults’ self-efficacy in performing activities of daily living, though it did not lead to measurable improvements in actual activity performance (35). Sharing this literature may help these staff members better understand the rationale behind the design of the activities of daily living exercise. Additionally, some staff commented that a rehabilitation health professional should deliver these daily activity exercises. These findings suggest that fitness staff may confine their role to the gym environment and are hesitant to extend their work into residents’ homes. Their primary focus remains on promoting fitness rather than re-ablement. Delivering activities of daily living exercises may push them out of their comfort zone. To address this, a campaign to expand the fitness role and emphasize the potential for reducing late-life disability in retirement communities may improve acceptance of delivering the activities of daily living exercise.

## 5 Strengths and limitations

To the best of current knowledge, no prior studies have explored retirement community fitness staff’s views on the feasibility of implementing evidence-based exercise programs to reduce late-life disability in at-risk residents. A major strength of this study is the use of semi-structured interviews to guide the fitness staff participants in discussing factors that may influence the implementation in depth, although the sample size appears to be limited. Nevertheless, the study was conducted during the COVID-19 pandemic, which may have affected the number of communities willing or able to participate in the study. Additionally, most fitness programs were adapted to comply with safety measures, often shifting to virtual formats, during the pandemic (36). This context might have influenced the fitness staff’s perceptions. Despite the limitations, the findings are critical for informing the future promotion of evidence-based programs in retirement communities to support age in place.

## 6 Conclusion

The fitness staff in the retirement community supported using the task-oriented resistance exercise program as a means to reduce late-life disability among at-risk residents. The screening process helps staff identify those who would benefit from the program while directing ineligible residents to alternative fitness resources. The small group format for resistance exercise allows for proper supervision and fosters camaraderie among at-risk residents. Furthermore, the one-on-one format for activities of daily living exercise encourages at-risk residents to engage in physical activity without leaving home and to see improvements in their daily tasks. The program’s duration, frequency, and intensity of exercises are considered appropriate to optimize benefits. However, concerns about implementation include constraints on fitness personnel, the staff’s current scope of work, and their comfort level when working alone with at-risk older adults in their homes. The next steps would be to collaborate with community stakeholders to address these barriers and build capacity to implement such a program.

## Data Availability

The raw data supporting the conclusions of this article will be made available by the authors, without undue reservation.
