# Supplementary material for: “I value it, but someone should do it”: fitness staff’s perspectives on task-oriented resistance exercise in retirement communities
Source: Front Public Health. 2026 Jan 8;13:1718945. doi: 10.3389/fpubh.2025.1718945 (PMC12823794; doi:10.3389/fpubh.2025.1718945)
Supplement: Supplementary file 1 [file Table_1.DOCX]

Appendix. Study Interview Guide

1. What is your age range: 20-29, 30-39, 40-49, 50-59, 60 or above?
2. What is your gender?
3. What is your ethnic background?
4. Please describe your work history and professional experience as a fitness instructor.

- What is your job title?
- What are your job responsibilities?
- How long have you been a fitness instructor [job title]?
- How long have you been working in a senior living community as a fitness instructor?
- How long have you been working in the current senior living community?
- Full-time or part-time?

1. Please describe the fitness environment in your community.

- Tell me about the fitness environment in [name of the senior living community]. How many gyms? How big are these gyms? What (equipment) is available in these gyms?
- Do you have fitness personnel in these gyms during the daytime to provide supervision?
- What fitness measures do you use to evaluate the residents’ fitness level or track their improvement?
- What group fitness classes does this community offer to its residents? How big and how long are these classes? What is the exercise intensity of these classes? Are these mostly chair exercises? Who developed these classes, or who provides these classes?
- Are these classes free to all residents? Do they need to sign up for each class, or is it first-come, first-serve?
- Do you offer group fitness classes? What are these classes you offered?
- Does the community offer an individualized fitness program (which is similar to personal training) to the residents? If so, tell me more about it. How big is the program? Is the program offered in the gym or the resident’s home? What are the program’s objectives?

1. Residents’ participation
   - - How large is your community? How many residents?
     - What percentage of residents in the community use the gym? Attend fitness classes?
     - What types of exercise or what classes are popular among the residents?
     - What do you or the community do to encourage the residents to exercise or stay active?
2. The task-oriented resistance exercise program

How do you feel if you need to conduct a screening and select eligible residents for this program, if this program were offered in your community? How do you think the residents would like to be screened and told that they are or are not eligible for this class? What would be their responses since most fitness classes do not require a screening and selection?

The task-oriented exercise program is a 10-week program, with one-hour sessions 3 times a week. Residents participating in this program would be expected to complete 30 one-hour fitness classes over a 10-week period.

- What do you think about the time commitment required for residents to participate in this program?
- Does this program’s duration and frequency affect your level of interest in delivering the program?

The program utilizes two types of exercise: resistance exercise and activities of daily living exercise. In the resistance exercise portion of the program, residents will use an elastic band to improve muscle strength. Most of these band exercises can be completed while seated. An instructor will lead these exercises, teaching 4-6 residents at a time. The class size is kept small so that the instructor can properly supervise each resident.

- What do you think about the class size? Is it appropriate?
- Are you expected to have bigger fitness classes? In other words, do you have productivity pressure to have a bigger class?
- What is your experience in teaching elastic band exercise?
- In your experience, can residents tolerate moderate exercise intensity (can do 12 repetitions of each movement for 2 or 3 sets, or stop when they feel slight muscle burn)?

The second exercise targeted in this program is activities of daily living exercise. In this part of the program, an instructor conducts one-on-one personalized exercise sessions in each resident’s home. Basically, the resident’s home becomes a gym. The participant’s household chores and tasks will be adapted for exercise purposes. For example, weight will be attached to a vacuum cleaner, and the resident will be asked to clean their living room floor. The daily living exercise will include a moderate intensity level.

- How feasible is it for you to perform one-on-one exercise sessions at a resident’s home, considering your responsibilities within the senior living community?
- How receptive would the residents be to having you conduct these exercise sessions in their homes?
- Are you comfortable instructing this type of exercise, asking the residents to perform housework as exercise?

I am going to summarize the instructor’s responsibilities and time commitment to give you a better insight into the program’s 10-week schedule. For the first half of the program, the instructor will lead one-hour-long group resistance band exercise sessions three days a week. For the second half of the program, the instructor will lead the elastic band exercises twice a week. The third day of resistance exercise will be replaced with the individualized activities of daily living exercise. These sessions will also last one hour; however, they will be held one-on-one in the participant’s home. So, if there are 6 participants, the instructor will need to lead the activities of daily living exercise for six hours on Friday.

- Knowing this now, how feasible is it for you to offer such exercise one-on-one at the resident’s homes in your community?
- What are the barriers for you and the residents to complete this part of the exercise?
- What are the potential benefits for you and the residents to complete this part of the exercise?

What kind of support would you need to implement this program?
